# MICA+ Tumor Cells Modulate Macrophage Phenotype and Function via PPAR/EHHADH-Mediated Fatty Acid Metabolism in Hepatocellular Carcinoma (HCC)

**DOI:** 10.3390/cancers17142365

**Published:** 2025-07-16

**Authors:** Jingquan Huang, Yumeng Teng, Peng Yan, Yan Yang, Shixun Lin, Qiulin Wu, Qiang Du, Xicai Li, Ming Yao, Jianjun Li, Yubin Huang, Xiaoyong Cai, David A. Geller, Yihe Yan

**Affiliations:** 1 Department of General Surgery, The Second Affiliated Hospital of Guangxi Medical University, Nanning 530007, China; 2 Department of Surgery, University of Pittsburgh Medical Center, Pittsburgh, PA 15260, USA

**Keywords:** MICA, macrophage, phenotype, PPAR, EHHADH, fatty acid oxidation, hepatocellular carcinoma

## Abstract

**Background:** Tumor-associated macrophages (TAMs) play a crucial role in the tumor microenvironment (TME), and the metabolic activities of both tumor cells and TAMs have an impact on the TME. Moreover, the expression of MICA in tumor cells is closely associated with immune cells in hepatocellular carcinoma (HCC). However, it remains unclear whether MICA expression correlates with TAMs and influences the switch in macrophage phenotype by mediating metabolic alterations. **Methods:** Various biostatistical tools, qPCR, and IHC staining experiments were utilized to analyze data from The Cancer Genome Atlas (TCGA) and collected HCC tumor tissues. Single-cell RNA sequencing (scRNA-seq) analyses and a co-culture model of HCC cells with macrophages were performed to validate the findings from the biostatistical analyses. **Results:** Through the intersection of differentially expressed genes (DEGs), metabolism-related genes (MRGs), and co-expression genes (CEGs) with MICA in HCC, the EHHADH gene was identified. Gene set enrichment analyses were conducted to further confirm the role of EHHADH. EHHADH expression is decreased in HCC tumors and can serve as a prognostic biomarker for HCC. Expressions of MICA and EHHADH exhibited significant correlations with various phenotypic macrophages and exerted opposing effects on M1-like and M2-like macrophages infiltrating HCC. The underlying metabolic and molecular mechanisms revealed that MICA in tumor cells induced M2-like polarization through the PPAR/EHHADH pathway, which regulates the fatty acid oxidation (FAO) in macrophages. **Conclusions:** The metabolic gene EHHADH, which is associated with MICA, led to alterations in M2-like macrophages by promoting heightened fatty acid uptake and augmenting levels of FAO within macrophages.

## Simple Summary

This study provides evidence that the MICA-related metabolic gene EHHADH has potential as a prognostic biomarker for HCC. MICA induces macrophage polarization toward an M1-like phenotype in early-stage HCC, which may have an antitumor effect. However, in late-stage HCC, MICA induces a decrease in EHHADH expression, leading to macrophage conversion to the anti-inflammatory M2-like phenotype, which may contribute to tumor progression. The increase in the utilization rate of fatty acids by macrophages promotes this transformation and enhances the energy production of fatty acid oxidation (FAO). Consequently, EHHADH emerges as a promising therapeutic target for HCC. Combining the induction of MICA (pro-inflammatory effect) in cancer cells with the specific inhibition of FAO (anti-inflammatory effect) in macrophages may offer a beneficial therapeutic approach for HCC.

## 1. Introduction

The investigation of the tumor microenvironment (TME) in hepatocellular carcinoma (HCC) has garnered significant interest in recent times. The TME assumes a pivotal role in the initiation and progression of HCC, establishing a conducive environment that facilitates cancer cell proliferation, metastasis, and dampens antitumor immune responses [1,2]. Within the HCC milieu, distinct immune cell populations exhibit diverse functions. Concurrently, cancer cells secrete a repertoire of cytokines that orchestrate tumor advancement by modulating immune cell behavior within the TME, ultimately promoting a tumor-supportive phenotype [3].

Macrophages, as innate immune cells, are widely distributed within the TME and hold significant importance in both autoimmunity and cancer [4]. These macrophages possess a high degree of plasticity and respond to changes in their surroundings by modulating cellular metabolism and immunophenotype. Typically, they can be categorized into two main subtypes, namely the classically activated M1-like macrophages and the alternatively activated M2-like macrophages [5]. M1-like macrophages, induced by lipopolysaccharide (LPS), exhibit a pro-inflammatory phenotype, crucial for tumor cell elimination and the secretion of pro-inflammatory cytokines. Conversely, M2-like macrophages, induced by interleukin (IL)-4 and IL-13, display an anti-inflammatory phenotype, secreting anti-inflammatory cytokines and promoting tissue remodeling and progression [6,7]. The dual role of macrophages may be the result of their interaction with T cells. For example, they can modulate the immune response of macrophages to pathogens, autoantigens, tumors, and transplant antigens through the CD40 ligand–CD40 interaction [8]. Within the TME, tumor-associated macrophages (TAMs) often adopt an M2-like phenotype, characterized by the secretion of anti-inflammatory cytokines, the promotion of tumor cell proliferation, and the suppression of antitumor immune responses [9].

Numerous investigations have been conducted to explore the intricate relationship between immune cell metabolism and function. In the context of malignancies, the metabolic profile of immune cells undergoes reprogramming, thereby influencing their biological behavior [10]. In the case of M1-like macrophages, their metabolic phenotype is characterized by heightened glycolysis, augmented flux through the pentose phosphate pathway (PPP), increased fatty acid synthesis (FAS), and inhibition of the tricarboxylic acid (TCA) cycle. Notably, M1-like macrophages primarily rely on glycolysis as their energy source. Conversely, M2-like macrophages exhibit reduced glycolysis and enhanced fatty acid oxidation (FAO). The energy requirements of M2-like macrophages predominantly rely on FAO-derived oxidative phosphorylation (OXPHOS) [11,12]. Consequently, modulating the metabolic pathways of macrophages holds the potential to induce phenotypic switching in these cells.

Major histocompatibility complex (MHC) class I polypeptide-related sequence A (MICA) serves as the ligand for natural killer group 2 member D (NKG2D), a protein encoded by the killer cell lectin-like receptor K1 (*KLRK1*). NKG2D is expressed on a wide range of immune cells, including NK cells, CD8+ T cells, NKT cells, and activated macrophages. Upon the binding of MICA to NKG2D, NKG2D engages with the adaptor dimer DAP10, initiating signaling activation, which promotes cell-mediated cytotoxicity, cytokine production, and the clearance of tumor cells [13,14].

MICA plays a crucial role in antitumor immune responses. Our previous investigation demonstrated a positive correlation between MICA expression and the infiltration of NK cells and CD8+ T cells in HCC [15]. Conversely, several researchers have observed that increased MICA expression in HCC and small-cell lung cancer (SCLC) is associated with poor prognosis [16,17]. This association may be attributed to the hydrolytic shedding of MICA mediated by the MMP9 secreted from TAMs in HCC according to our subsequent study [18]. However, the association between MICA expression and TAMs in HCC remains unclear. Additionally, cellular metabolism has been shown to influence macrophage function. Previous studies have suggested a connection between MICA and cellular metabolism [19,20]. Nevertheless, the precise mechanism by which MICA modulates macrophage phenotype through mediating metabolic alterations in HCC remains elusive. Therefore, there is an urgent need to investigate the molecular mechanisms underlying the impact of tumor cell-derived MICA on the metabolic and phenotypic alterations of macrophages in HCC.

In this study, we conducted an analysis utilizing data from The Cancer Genome Atlas (TCGA) and employed various tools, including Gene Ontology (GO), the Kyoto Encyclopedia of Genes and Genomes (KEGG), and receiver operating characteristic (ROC) curves, to identify and select the target gene, namely Enoyl-CoA Hydratase And 3-Hydroxyacyl CoA Dehydrogenase (EHHADH), a gene associated with metabolism that exhibits co-expression with MICA in HCC. Subsequently, we utilized gene expression profiling interaction analysis (GEPIA), the cBio Cancer Genomics Portal (cBioportal), and the Tumor Immune Estimation Resource (TIMER) to assess the expression levels and prognostic significance of EHHADH. We further explored the association between EHHADH, MICA, and phenotypic markers of macrophages through biostatistical analyses and experimental validation using clinical tumor tissue obtained from HCC patients. Additionally, we investigated the expression of EHHADH and its upstream gene, peroxisome proliferator-activated receptor (PPAR), on various cells within the TME using single-cell RNA sequencing (scRNA-seq) analyses. We found that PPAR-α regulated EHHADH expression in HCC cells and that this signaling pathway was suppressed in the late-stage HCC. To confirm the role of MICA in tumor cells in inducing M2-like polarization through the PPAR-α/EHHADH pathway, we employed a co-culture model consisting of MICA+HCC cells and macrophages. This model allowed us to examine the regulation of FAO in both tumor cells and macrophages.

## 2. Materials and Methods

### 2.1. Data Collection

UCSC Xena (https://xenabrowser.net/) is an online site that was utilized to access secondary developed TCGA data [21]. Specifically, FPKM-format transcriptome data and clinical information files pertaining to HCC in TCGA were obtained from UCSC Xena. This dataset encompassed 374 HCC tumor tissue samples and 50 adjacent normal tissue samples.

To identify metabolism-related genes (MRGs), GeneCards (RRID:SCR_002773) (https://www.genecards.org/), an integrative database [22], was employed. A search using the keyword “metabolism” was conducted, and genes with a correlation score greater than 5 were considered as MRGs.

Furthermore, the cBioPortal (http://www.cbioportal.org/), a freely accessible database that consolidates research data from various sources, including TCGA [23], was utilized. The list of co-expression genes (CEGs) associated with MICA in HCC was downloaded from the cBioPortal. Genes with an absolute correlation value of 0.2 or higher were deemed as CEGs.

### 2.2. Screening for Differentially Expressed Genes (DEGs)

To identify DEGs, all transcriptome data from the HCC cohort in TCGA were subjected to analysis using the LIMMA (RRID: SCR_010943, version: 3.48.3) R package [24]. DEGs were defined as genes exhibiting a false discovery rate (FDR) of less than 0.05 and an absolute log fold change (|logFC|) greater than 1.

### 2.3. Screening of Target Genes and Functional Enrichment Analysis

The intersection of MRGs, CEGs, and DEGs was selected as the pool of genes co-expressed with MICA in HCC for subsequent analysis. To gain insights into the functional implications of these genes, GO analysis and KEGG (RRID: SCR_012773) pathway analysis were conducted using the clusterProfiler (RRID: SCR_016884) R package [25]. Statistical significance was determined by a *p*-value threshold of less than 0.05.

### 2.4. Expression and Prognostic Analysis of EHHADH

To explore the expression and prognostic significance of EHHADH in HCC, we utilized the GEPIA online tool (http://gepia.cancer-pku.cn/index.html (accessed on 18 November 2022)), which enables bioinformatic analyses of data from the TCGA and Genotype–Tissue Expression (GTEx) databases [26]. Firstly, the correlation between EHHADH expression and clinicopathological information in HCC was assessed using GEPIA. Additionally, the differential expression of EHHADH between HCC tumor tissue and normal tissue was determined. Moreover, the levels of EHHADH expression across different pathological stages were compared. Furthermore, the association between EHHADH expression and prognosis in HCC was evaluated.

To complement our analysis, we utilized the Human Protein Atlas (HPA) database (https://www.proteinatlas.org/), which provides information on protein expression in normal tissues, tumor tissues, and cells [27]. The protein expression of EHHADH was explored using the HPA database. To assess the predictive accuracy of EHHADH expression for prognosis in HCC, we constructed receiver operating characteristic (ROC) curves using the pROC R package (version: 1.0-11).

### 2.5. Immune Cell Infiltration Analysis

The TIMER (https://cistrome.shinyapps.io/timer/ (accessed on 3 April 2022)) and TIMER2.0 (http://timer.comp-genomics.org/ (accessed on 24 March , 2022)) databases were used to assess immune cell infiltration in various cancer types [28,29]. Specifically, we obtained information on the levels of EHHADH mRNA in pan-cancer (33 cancer types) and investigated the relationship between EHHADH expression in HCC and tumor purity as well as immune infiltrating abundance using TIMER. Furthermore, the association between immune infiltration and copy number variation (CNV) of the EHHADH gene was analyzed using TIMER. Additionally, we obtained information on the association between EHHADH expression and tumor purity, as well as the immune-infiltrating abundance of M1-like or M2-like macrophages, through the utilization of TIMER2.0.d via TIMER2.0.

### 2.6. Cancer Genomics Analysis of the cBioPortal Database

Co-expression relationships among genes in patients with HCC were accessed by using the cBioPortal database.

### 2.7. The Acquisition of scRNA-Seq Data

To obtain scRNA-seq data for our study, we downloaded the data from the GEO database (https://www.ncbi.nlm.nih.gov/geo (accessed on 10 November 2022)) for five samples (GSM4505944, GSM4505945, GSM4505953, GSM4505961, and GSM4505964) of GSE149614. In total, we obtained data from 17,392 cells and 25,712 genes across the 5 patients. Relevant clinical information of the five patients is provided in Table S1. Among these patients, two were diagnosed with AJCC stage I, one was diagnosed with stage IIIA, and three were diagnosed with stage IV, all of whom had a background of hepatitis [30].

### 2.8. Preprocessing and Analysis of scRNA-Seq Data

The scRNA-seq data obtained from the previous section were subjected to preprocessing and analysis using the R software “SEURAT (RRID:SCR_007322)” package (v4.2.0). To ensure the retention of high-quality data, raw matrices underwent filtering to remove cells with less than 200 transcripts per cell and more than 15% mitochondrial genes, as well as genes with less than 3 cells expressing them. The “NormalizeData” function of the “Seurat” package was then applied to the data, with the normalization method set as “LogNormalize”.

Principal component analysis (PCA) was performed using the Seurat package in R software (v4.2.1), along with the JackStraw and ElbowPlot functions, to select the top 20 principal components (PCs). Cell clustering analysis was conducted using the “FindNeighbors” and “FindClusters” functions in the Seurat package. Subsequently, cell clustering and visual analysis were performed using the “RunTSNE” and “RunUMAP” functions.

To compare differences in gene expression between clusters, the “FindAllMarkers” function was utilized. Marker genes for each cluster were identified based on an adjusted *p*-value < 0.05 and |log2(fold change)| > 1. The annotation of cell clusters was based on marker genes from the CellMarker (RRID: SCR_018503) databases and genes reported in the literature [31].The marker genes of each cell cluster are provided in Table S2.

### 2.9. Patient Sample

Clinical HCC tumor tissue and adjacent liver samples were obtained from 69 HCC patients who underwent hepatectomy at the Second Affiliated Hospital of Guangxi Medical University. All human tissues were obtained according to protocols (No. 2022-KY-0208) approved by the Ethics Committee of Second Affiliated Hospital of Guangxi Medical University. Prior informed consent was obtained from the patients.

### 2.10. Quantitative Real-Time PCR (qRT-PCR)

Total RNA was extracted using TRIzol reagent (New Cell & Molecular Biotech, Suzhou, China). RNA was reverse-transcribed into cDNA using cDNA synthesis kit (A230, GenStar, San Francisco, CA, USA). Subsequent qRT-PCR was performed using a PCR kit (A303, GenStar), and relative expression was calculated by the 2^−ΔΔCt^ method [32]. Primer sequences used in this study were provided in Table 1.

### 2.11. Immunohistochemistry (IHC)

Formalin-fixed and paraffin-embedded sections were subjected to de-paraffinization using xylene followed by rehydration. Heat-mediated antigen retrieval was performed using citrate buffer (pH = 6). Subsequently, the sections were incubated overnight at 4 °C with the following primary antibodies: anti-EHHADH (1:400, Proteintech, Wuhan, China,, Cat# 26570-1-AP), anti-CD86 (1:200, Affinity Biosciences, Zhenjiang, China, Cat# DF6332), anti-CD206 (1:1000, Proteintech Cat# 60143-1-Ig), and anti-MICA (1:500, Proteintech Cat# 66384-1-Ig). Afterward, the sections were incubated with a secondary antibody (PV9000, ZSGB-BIO, Shanghai, China) for 20 min. Staining was visualized using 3,3′-diaminobenzidine solution (DAB) (ZLI-9017, ZLI-9018, ZLI-9019, ZSGB-BIO), and counterstained with hematoxylin (G1080, Solarbio, Beijing, China). The sections were then subjected to hydration, dehydration, and sealing. Finally, the staining intensity was quantified by calculating the average optical density (AOD) values using Fiji (RRID: SCR_002285, version: 2018-11-30) software [33,34].

### 2.12. Cell Culture

The mouse Hepa 1-6 and human Huh-7 (RRID: CVCL_0336) and HepG2 (RRID: CVCL_0027) hepatic cancer cells and the THP-1 (RRID: CVCL_0006) macrophage cells were purchased from the Shanghai Institute of Biochemistry and Cell Biology, Chinese Academy of Sciences. HCC cell lines were cultured in Dulbecco’s modified Eagle’s medium (C11995500BT, Thermo Fisher, Waltham, MA, USA), while THP-1 cells were cultured in RPMI 1640 Medium (C11875500BT, Thermo Fisher). The medium was supplemented with 10% heat-inactivated fetal bovine serum (FBS) and 100 U/mL penicillin, and 100 mg/mL streptomycin (C125C5, New Cell & Molecular Biotech). All cell lines were cultured at 37 °C in a humidified atmosphere of 5% CO_2_.

### 2.13. Lentivirus Infection

A lentiviral vector carrying human MICA cDNA or empty vector was constructed by Genechem (Shanghai, China). According to the standardized protocols, HCC cells were seeded, and the following day they were infected with lentivirus for 48 h according to the experimental MOI. Next, the culture medium was changed, and the cells stably expressing *MICA* gene were sorted out by flow cytometry and expanded. Clones were subsequently screened by qRT-PCR and Western blotting for MICA mRNA and protein expression.

### 2.14. Western Blotting

Cells were treated with RIPA cell lysate supplemented with 1% PMSF. Subsequent centrifugation was performed to collect the supernatant and to obtain proteins. The proteins were then mixed with 5× sampling buffer and subjected to boiling. Next, 30 µg of protein solution was loaded onto a sodium dodecyl sulfate–polyacrylamide gel for electrophoresis. Following electrophoresis, the proteins were transferred onto a 0.45 µm polyvinylidene difluoride membrane. The membrane was then blocked with a 5% BSA blocking solution for 1 h. Incubation with primary antibodies was carried out overnight at a dilution of 1:1000. The primary antibodies used were anti-EHHADH (1:1500, Proteintech Cat# 26570-1-AP), anti-MICA (1:2000, Proteintech Cat# 66384-1-Ig), and anti-GAPDH (1:5000, Solarbio Cat# K200057M). After washing the membrane three times with Tris-buffered saline and Tween-20 (TBST), it was incubated with fluorescent secondary antibodies diluted 1:20,000 for 1 h. The membrane was then scanned using an Odyssey imaging system (Odyssey CLX, LI-COR, Lincoln, NC, USA) [35].

### 2.15. Co-Culture

THP-1 cells were seeded at a density of 1 × 10^5^ cells per well into a six-well plate and treated with 100 nm phorbol 12-myristate 13-acetate (PMA) (MedChemExpress, Monmouth Junction, NJ, USA) for 24 h. This treatment led to the differentiation of THP-1 cells into macrophages. Following a change in the culture medium, the macrophages were inoculated into six-well plates and co-cultured with MICA+HCC cells or negative control (NC) HCC cells, which were inoculated at a density of 1 × 10^5^ cells per well in the upper six-well Transwell device (0.4 μm pore size, 3412, Corning, Kenneburg, ME, USA), for either 24 or 72 h. To inhibit PPAR-α activity, macrophages in the relevant groups were treated with GW6471 (10 µm, HY-15372, MedChemExpress) before co-cultivation [36]. Next, both HCC cells and macrophages were washed and collected for subsequent experiments.

### 2.16. Immunofluorescence (IF) Staining

Coverslips were positioned within six-well plates. THP-1-derived macrophages, obtained after co-culture, were collected and seeded into six-well plates with coverslips. These cells were then cultured for a duration of 24 h. Following this, they were fixed using 4% paraformaldehyde, permeabilized with 0.5% Triton X-100 (T8200, Solarbio), and subsequently blocked with 5% goat serum (ZLI-9021, ZSGB-BIO). Subsequently, the macrophages were incubated with anti-EHHADH (1:200, Proteintech Cat# 26570-1-AP), anti-CD86 (1:500, Affinity Biosciences Cat# DF6332), anti-CD206 (1:500, Proteintech Cat# 60143-1-Ig), anti-IL-10 (1:300, Proteintech Cat# 60269-1-Ig), and anti-TNF-α (1:300, Proteintech Cat# 60291-1-Ig) antibodies at 4 °C overnight. They were then incubated with secondary antibodies (1:1000, Cell Signaling Technology, Danvers, MA, USA; 1:1000, Proteintech Cat# SA00013-1). Finally, the cell nuclei were stained using DAPI-containing anti-fluorescence quenching encapsulants (Seven Biotech, Worcestershire, UK) [37]. The fluorescence intensity was subsequently quantified using ImageJ, RRID: SCR_003070 software (version: 1.53K) [38].

### 2.17. Oil Red O Staining

The extent of lipid accumulation in HCC cells and macrophages following co-culture was evaluated using Oil Red O staining. Cells obtained after co-culture were seeded into a 96-well plate. Subsequently, the cells were treated with 250 μm of free fatty acid oleate (oleic acid–palmitic acid ratio of 2:1) for a duration of 24 h. After this treatment, the cells were fixed using 4% paraformaldehyde for 30 min and then incubated with 50 μL of Oil Red working solution (prepared by mixing Oil Red O stock solution with ddH_2_O in a 6:4 ratio) for 15 min. Following the incubation, the Oil Red O solution was removed, and the cells were washed with ddH_2_O until all excess stains were completely removed. The oil red dye was subsequently washed off using 100 μL of DMSO. The resulting lipid accumulation data were measured using a fluorescence microplate spectrophotometer at a wavelength of 510 nm [39].

### 2.18. BODIPY Staining

The level of lipid accumulation in HCC cells and macrophages after co-culturing was assessed by BODIPY staining. Cells were fixed with 4% paraformaldehyde for 15 min. Next, cells were stained with 10 μm BODIPY 493/503 (HY-W090090, MedChemExpress) for 20 min. Subsequently, cell nuclei were stained with DAPI-containing anti-fluorescence quenching encapsulants (Seven Biotech) [40]. Finally, fluorescence intensity was quantified using ImageJ software (version: 1.53K) [38].

### 2.19. FAO Testing

The ACADVL, ACADM, and HADHA expression in FAO pathway were measured with a commercial colorimetric assay kit (ab118182, Abcam, Boston, MA, USA) according to the manufacturer’s protocol [41].

### 2.20. ELISA Validation

Cell culture supernatants were collected for subsequent assays. Levels of TNF-α and IL-10 were, respectively, measured with ELISA kits (ml064303V and ml064299V, Mlbio; YJ077385 and YJ028605, Shanghai, China) according to the manufacturer’s protocol.

### 2.21. Cell Proliferation Assay

HCC cells were collected for subsequent experiments. Then, 100 μL of medium containing 1000 cells was inoculated into 96-well plates. Medium without cells was inoculated at the same time as blank control wells. Cells were incubated for 0 h, 24 h, 48 h, 72 h, and 92 h. Subsequently, 10 μL of CCK-8 solution was added to each well, and incubation was continued for 2 h. Finally, cell proliferation data were measured by a fluorescence microtiter spectrophotometer at a wavelength of 450 nm. Data were collected and subsequently calculated.

### 2.22. Apoptosis Assay

HCC cells were collected for subsequent experiments. Cells were lysed using 50 μL lysis buffer containing 0.5 μL DTT. After centrifugation of the supernatant, the protein content was detected. Subsequently, 50 μL of supernatant containing 100 μg of protein was added to a 96-well plate and incubated with 50 μL of 2× reaction buffer containing 0.5 μL DTT and 5 μL of caspase-3 substrate for 4 h. Subsequently, apoptosis data were measured by fluorescence microtiter spectrophotometer at a wavelength of 405 nm. Data were collected and subsequently calculated.

### 2.23. Statistical Analysis

Statistical analyses were conducted utilizing R (version 4.2.1) and SPSS, RRID: SCR_002865 (version 22.0). A significance level of *p* < 0.05 (two-sided) was employed to determine statistical significance for all analyses. Additionally, the TCGA data analysis online platform Sangerbox (http://vip.sangerbox.com/home.html (accessed on 26 October 2022)) was utilized for data analysis and visualization purposes [42]. In addition, we also used the Xiantao Academic Website (https://www.xiantaozi.com/) for bioinformatics analysis and image drawing.

## 3. Results

### 3.1. EHHADH Was Identified as a Candidate Gene Through the Integration of DEGs, MRGs, and CEGs with MICA in HCC

**Figure 1:**
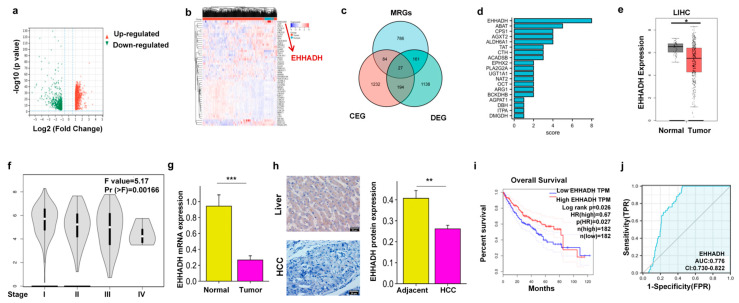
Screening, enrichment analysis, expression, and prognostic value analysis of the target gene EHHADH in HCC. (**a**) Volcano plot of DEGs of HCC patients in TCGA. Genes with FDR < 0.05 and |logFC| > 1 were considered as DEGs. Green: downregulated genes, red: upregulated genes. (**b**) Heat map of the top 50 DEGs. (**c**) Venn diagram of 27 metabolism-related genes co-expressed with MICA in HCC. (**d**) Frequency of gene occurrence in KEGG pathway enrichment analysis. (**e**) Differential expression of EHHADH between HCC and normal tissues. (**f**) Differential expression of EHHADH in different tumor stages of HCC. (**g**) EHHADH mRNA expression in HCC tumor tissue and normal (*n* = 32). (**h**) IHC staining for EHHADH in collected tumor and normal tissue from HCC patients (*n* = 31). Scale bars are 20 µm. (**i**) K-M survival analysis of EHHADH in HCC. (**j**) ROC curve of EHHADH in HCC. *p*-value significance codes: * *p* < 0.05, ** *p* < 0.01, and *** *p* < 0.001.

To identify DEGs, transcriptome data in the FPKM format from TCGA HCC patients obtained from the UCSC Xena database were analyzed. A total of 1555 DEGs were identified and visualized in a volcano plot (Figure 1a). The top 50 DEGs were further depicted in a heat map (Figure 1b). Additionally, GeneCards analysis revealed 1058 MRGs with a correlative score greater than 5. Furthermore, cBioportal analysis identified 1537 CEGs with an absolute correlation value of 0.2 or higher. By intersecting the DEGs, MRGs, and CEGs, we identified 27 metabolism-related genes that exhibited co-expression with MICA in HCC (Figure 1c).

Subsequently, functional enrichment analyses were conducted on the identified set of 27 genes using GO and KEGG databases. In terms of biological processes (BP), these genes were primarily associated with small molecule catabolic processes, carboxylic acid catabolic processes, and organic acid catabolic processes (Figure S1a). Analysis of cellular components (CC) revealed enrichment in the mitochondrial matrix, cytoplasmic vesicle lumen, and secretory granule lumen (Figure S1b). In relation to molecular function (MF), these genes were predominantly involved in coenzyme binding, organic acid binding, and carboxylic acid binding (Figure S1c). KEGG pathway enrichment analysis indicated that the intersecting genes were primarily associated with valine, leucine, and isoleucine degradation, biosynthesis of amino acids, and propanoate metabolism (Figure S1d). Notably, the EHHADH gene was found to be present in the most frequently occurring signaling pathways based on KEGG pathway enrichment analysis (Figure 1d). Consequently, among the 27 metabolic genes co-expressed with MICA in HCC, the EHHADH gene was selected for further investigation.

Given the functional relevance of EHHADH as an enzyme involved in FAO [43,44], our initial investigation focused on examining the differential expression of EHHADH mRNA in pan-cancer samples (33 cancer types) compared to normal tissues. Notably, we observed a significant decrease in EHHADH mRNA levels specifically in HCC tumors when compared to normal tissues (Figure S1e and Figure 1e). Furthermore, we found a pronounced decrease in EHHADH mRNA expression in advanced tumor stages (Figure 1f). Consistently, our analysis of collected samples confirmed a decreased EHHADH mRNA expression in HCC tumors compared to background liver tissues (Figure 1g). To complement our findings, we also examined EHHADH protein expression using IHC staining from the Human Protein Atlas (HPA) database. Interestingly, we observed high EHHADH expression in normal liver tissue and moderate expression in HCC tumors (Figure S1f). Moreover, our analysis of our collected HCC samples further validated the decreased EHHADH protein levels in tumors (Figure 1h).

Furthermore, we performed an analysis of DEGs in HCC samples with high and low EHHADH expression levels. A total of 456 DEGs were identified, with 285 being upregulated and 171 being downregulated. To gain further insights into the functional implications of these DEGs, we performed GO and KEGG pathway analyses. In terms of BP, the enriched categories included small molecule catabolic process, organic acid biosynthetic process, and fatty acid metabolic process. For CC, the significant categories involved blood microparticles, immunoglobulin complexes, and cytoplasmic vesicle lumens. In relation to MF, the identified categories were primarily associated with monooxygenase activity, antigen binding, and oxidoreductase activity, specifically acting on CH-OH group donors. Additionally, the KEGG pathway analysis revealed a significant correlation with metabolism of xenobiotics by cytochrome P450, chemical carcinogenesis, and the PPAR signaling pathway (Figure S1g).

To assess the potential of the EHHADH gene as a prognostic biomarker in HCC, we conducted Kaplan–Meier survival analysis. Our findings revealed that HCC patients with high EHHADH mRNA levels exhibited a significantly improved overall survival (OS) compared to those with low levels (hazard ratio [HR] = 0.67, *p* < 0.05; Figure 1i). Furthermore, we performed receiver operating characteristic (ROC) curve analysis to evaluate the predictive accuracy of EHHADH. The area under the curve (AUC) for EHHADH was determined to be 0.776 (95% confidence interval [CI]: 0.730–0.822; Figure 1j), indicating that EHHADH holds promise as a prognostic biomarker for HCC.

### 3.2. The Relationship Between MICA and EHHADH Was Associated with Macrophage Infiltration and Its Phenotype in HCC

Although our previous study demonstrated a positive correlation between MICA expression and infiltration of NK cells and CD8+ T cells in HCC [15], some studies showed that higher MICA expression in HCC was connected with unfavorable prognosis [17] and that antitumor immunity was attenuated by hydrolytic shedding of MICA mediated by macrophages [18]; thus, we subsequently investigated the relationship between MICA and EHHADH with macrophages.

Initially, we confirmed that higher MICA expression in HCC predicted poor prognosis through the TCGA database (Figure S2a). We also found no obvious alteration in the proliferation and apoptosis of MICA+ HCC cells (Figure S2b–d), which illustrated that MICA+ HCC cells probably posed an effect on HCC through regulating the immune microenvironment. Thus, we proceeded to investigate the impact of EHHADH on the immune microenvironment of HCC. We assessed the correlation between EHHADH expression, tumor purity, and the infiltration of immune cells in HCC using the TIMER database. Our analysis revealed a significant negative correlation between EHHADH mRNA expression and the infiltration of five immune cell types, namely B cells, CD8+ T cells, CD4+ T cells, neutrophils, and macrophages (Figure S3a). Furthermore, we observed a link between the copy number variation (CNV) of EHHADH and the infiltration of immune cells within the TME (Figure S3b).

Subsequently, we sought to investigate the correlation between EHHADH expression and distinct macrophage phenotypes. To accomplish this, we utilized the TIMER 2.0 database to analyze the relationship between EHHADH expression, tumor purity, and macrophage infiltration. Our analysis revealed a significant negative correlation between EHHADH expression and the infiltration of M1-like macrophages, while a positive correlation was observed with M2-like macrophages (Figure S3c,d). Interestingly, we also observed a positive correlation between MICA expression and macrophage infiltration in HCC (Figure S3e).

Given the co-expression of MICA and EHHADH in HCC (Figure 1c), as well as their significant correlation with macrophage infiltration in HCC (Figure S3c–e), our subsequent investigation focused on examining the correlation of EHHADH and MICA with classical phenotypic markers of macrophages using the cBioportal database. Initially, we observed a significant negative correlation between EHHADH mRNA level and MICA expression (Figure S4a). Furthermore, our analysis revealed a significant negative correlation between EHHADH expression and the levels of CD68 and ITGAM (CD11b), which are considered overall markers of macrophages [45] (Figure S4b,c), consistent with previous findings from TIMER. Additionally, EHHADH expression exhibited a clear negative correlation with the levels of CD86 and CD80, markers of M1-like macrophages [46] (Figure S4d,e), indicating a negative association between EHHADH and M1-like macrophage infiltration. Conversely, EHHADH expression displayed a significant positive correlation with the level of CD206 (MRC1), a marker of M2-like macrophages [47] (Figure S4f). Furthermore, we analyzed the correlation between MICA expression and macrophage markers. As expected, MICA expression positively correlated with CD68 and CD86, but negatively correlated with CD206 (Figure S4g–i).

**Figure 2:**
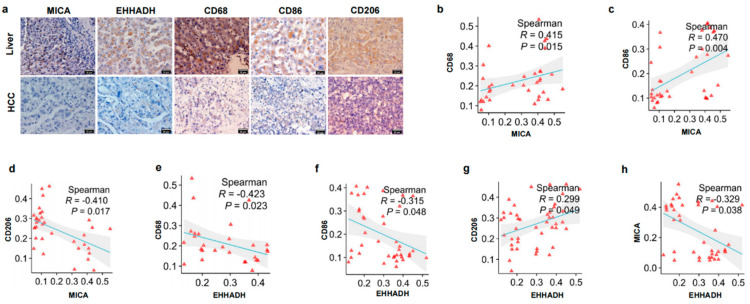
The IHC staining to verify correlation of MICA and EHHADH and macrophage infiltration in HCC. (**a**) Representative IHC staining for MICA, EHHADH, CD68, CD86, and CD206 in tumor and adjacent liver tissue from HCC patients (*n* = 36). All images were taken at 400× magnification. Scale bars are 20 µm. (**b**) Correlation of MICA protein expression with CD68 level in HCC tumor and background liver tissues (*n* = 22). (**c**) Correlation of MICA protein expression with CD86 level in HCC tumor and background liver tissues (*n* = 18). (**d**) Correlation of MICA protein expression with CD206 level in HCC tumor and background liver tissues (*n* = 21). (**e**) Correlation of EHHADH protein expression with CD68 level in HCC tumor and background liver tissues (*n* = 19). (**f**) Correlation of EHHADH protein expression with CD86 level in HCC tumor and background liver tissues (*n* = 20). (**g**) Correlation of EHHADH protein expression with CD206 level in HCC tumor and background liver tissues (*n* = 22). (**h**) Correlation of EHHADH protein expression with MICA level in HCC tumor and background liver tissues (*n* = 20).

To validate the findings from our biostatistical correlative analyses, we conducted qPCR and IHC staining experiments using the HCC tissue samples that we collected. Our results demonstrated a positive correlation between MICA mRNA and protein expression with the levels of CD68 and CD86, while a negative correlation was observed with CD206 (Figure S5a–c and Figure 2a–d). Conversely, EHHADH mRNA and protein expression exhibited a negative correlation with CD68 and CD86 levels, but a positive correlation with CD206 (Figure S5d–f and Figure 2e–g). Notably, the mRNA and protein expression of EHHADH showed a negative correlation with MICA (Figure S5g and Figure 2h). These experimental findings were consistent with the analyses conducted using the cBioportal database. Overall, our results indicated that MICA and EHHADH have opposing effects on the infiltration of M1-like and M2-like macrophages in HCC.

### 3.3. MICA+ HCC Cells Regulated Macrophage Polarization

**Figure 3:**
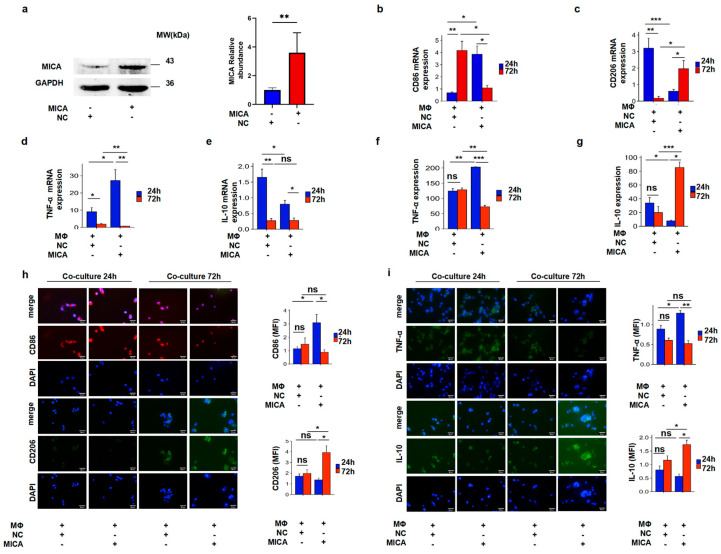
Co-culture of HCC cells with macrophage assays to verify the correlation between MICA and macrophage polarization. (**a**) Western blotting assay confirms increased MICA level in Huh-7 cells with stably over-expressed MICA compared to the control vector (NC); the statistically quantificational results are shown on the right (*n* = 4). The uncropped blots are shown in Supplementary File S1. (**b**) The CD86 mRNA expression is shown in macrophages co-cultured with MICA+ Huh-7 cells or NC cells for 24 h or 72 h (*n* = 9). (**c**) The CD206 mRNA expression is shown in macrophages co-cultured with MICA+ Huh-7 cells or NC cells for 24 h or 72 h (*n* = 8). (**d**) The TNF-α mRNA expression is shown in macrophages co-cultured with MICA+ Huh-7 cells or NC cells for 24 h or 72 h (*n* = 8). (**e**) The IL-10 mRNA expression is shown in macrophages co-cultured with MICA+ Huh-7 cells or NC cells for 24 h or 72 h (*n* = 6). (**f**) The TNF-α levels in supernatants from cultured macrophages were measured by ELISA (*n* = 5). (**g**) The IL-10 levels in supernatants from cultured macrophages were measured by ELISA (*n* = 9). (**h**) Representative immunofluorescence staining for CD86 (*n* = 4) and CD206 (*n* = 5) in macrophages co-cultured with MICA+ Huh-7 cells or NC cells for 24 h or 72 h and statistically quantificational results. (**i**) Representative immunofluorescence staining for TNF-α (*n* = 3) and IL-10 (*n* = 6) in macrophages co-cultured with MICA+ Huh-7 cells or NC cells for 24 h or 72 h and statistically quantificational results. All images were taken at 400× magnification. *p*-value significance codes: * *p* < 0.05, ** *p* < 0.01, and *** *p* < 0.001.

To investigate the potential correlation between MICA and EHHADH expression and the polarization of macrophages in HCC, we initially established HCC cells with stable expression of MICA (Figure 3a). We performed a co-culture experiment involving HCC cells and macrophages. Furthermore, we observed an increase in CD86 mRNA levels and a decrease in CD206 levels in macrophages co-cultured with MICA+HCC cells for 24 h. However, the expression of CD86 and CD206 mRNA reversed after 72 h of co-culture (Figure 3b,c). Additionally, we noted an increase in the mRNA level of the pro-inflammatory cytokine TNF-α and a decrease in the mRNA level of the anti-inflammatory cytokine IL-10 in macrophages co-cultured for 24 h. Conversely, this alteration was reversed in macrophages co-cultured for 72 h (Figure 3d,e). Furthermore, we validated these findings through ELISA and IF staining assays for the corresponding markers (Figure 3f–i). Thus, MICA+ HCC cells induced M2-like macrophages polarization along with the decreased EHHADH expression in the late stage.

### 3.4. MICA+ HCC Cells Increased Fatty Acid Accumulation Through the Decreased PPAR-α/EHHADH Signaling Pathway

**Figure 4:**
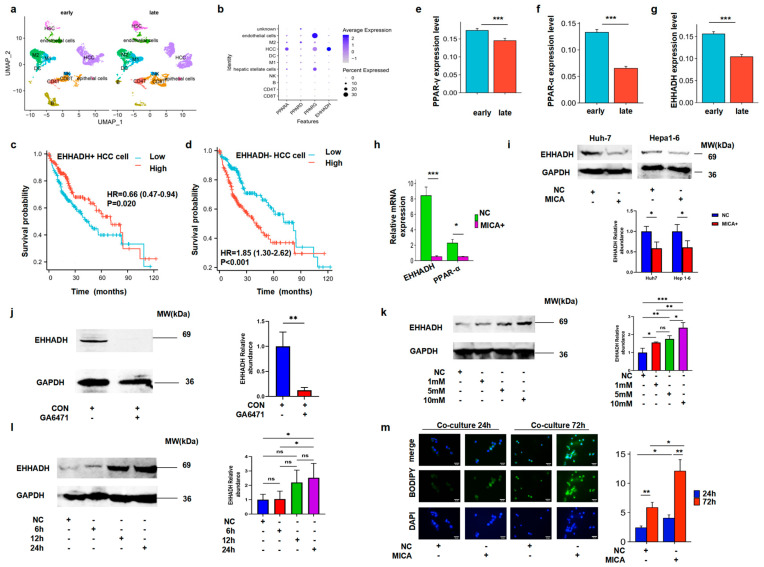
MICA+ HCC cells downregulated FAO through the decreased PPAR-α/EHHADH signaling pathway. (**a**) Different types of cells in HCC at the early and late stages are shown. (**b**) PPAR-α, -β, -γ, and EHHADH mRNA expression is shown on different types of cells in HCC. (**c**) K-M survival analysis shows that patients with fewer EHHADH+ cancer cells were associated with worse overall survival in the TCGA-LIHC patients (*n* = 371). (**d**) K-M survival analysis show that patients with more EHHADH- cancer cells were associated with worse overall survival in the TCGA-LIHC patients (*n* = 371). (**e**–**g**) PPAR-γ, PPAR-α, and EHHADH mRNA expression is shown in early-stage HCC cells compared to late-stage cells. (**h**) EHHADH (*n* = 9) and PPAR-α (*n* = 6) mRNA expression is shown in MICA+ Huh-7 cells compared to NC+ Huh-7 cells. (**i**) Western blotting confirms decreased EHHADH expression in MICA+ Huh-7 cells (*n* = 3) and MICA+ Hepa1-6 cells (*n* = 3) compared with NC cells, respectively. The statistical results are shown below. (**j**) Western blotting assay confirmed a decrease in EHHADH in PPAR-α inhibitor-treated Huh-7 cells compared to control cells. The statistically quantificational results are shown on the right (*n* = 3). (**k**) Western blotting assay confirmed an increased EHHADH expression in PPAR-α agonist-treated Huh-7 cells for 24 h with an increased dose compared to control cells. The statistically quantificational results are shown on the right (*n* = 3). (**l**) Western blotting assay confirmed an increased EHHADH expression in PPAR-α agonist-treated Huh-7 cells with increased treatment time compared to control cells. The statistically quantificational results are shown on the right (*n* = 5). The uncropped blots are shown in Supplementary File S1. (**m**) Representative BODIPY staining for co-cultured Huh-7 cells for 24 h and 72 h and associated statistical plots (*n* = 9). All images were taken at 400× magnification. *p*-value significance codes: * *p* < 0.05, ** *p* < 0.01, and *** *p* < 0.001.

Since our previous research based on scRNA-seq data demonstrated the predominant expression of MICA in HCC tumor cells [15], the co-expression of MICA and EHHADH in HCC (Figure 1c), and the confirmation of the significant correlation between MICA and EHHADH expression with macrophage infiltration in HCC (Figures S3–S5 and Figure 2), as well as the negative correlation between EHHADH and MICA (Figures S4a and S5g and Figure 2h), we proceeded to investigate the cell clusters in which EHHADH was expressed. By utilizing scRNA-seq data from the GEO database, we identified distinct cell types in early- and late-stage HCC (Figure 4a). Interestingly, EHHADH expression was predominantly localized to HCC cells (Figure 4b). Importantly, HCC patients with low EHHADH expression in cancer cells predicted poor prognosis (Figure 4c,d).

In the KEGG pathway analysis, the PPAR pathway was a lipid metabolism -elated pathway. Given that EHHADH was found to be enriched in the PPAR signaling pathway (Figure S1g), we further examined the expression of PPAR on HCC cells. PPAR-α and PPAR-γ, two subtypes of PPAR, were consistently primarily expressed on HCC cells, mirroring the expression pattern of EHHADH (Figure 4b). Importantly, the mRNA expression levels of PPAR-α, PPAR-γ, and EHHADH were found to be decreased in late-stage HCC cells compared to early-stage cells (Figure 4e–g). Our own qPCR experiments on collected HCC samples have also confirmed this (Figure S6a–c).

Subsequently, we confirmed a decrease in EHHADH and PPAR-α mRNA levels in MICA+ HCC cells (Figure 4h). Also, we confirmed the decreased EHHADH protein expression in MICA+ HCC cells by WB and IF staining assays, respectively (Figure 4i and Figure S7a). Additionally, we found decreased EHHADH protein expression in HCC cells with inhibited PPAR-α level (Figure 4j). PPAR-α agonist induced EHHADH expression in HCC cells in a dose- and time-dependent manner (Figure 4k,l).

Since PPAR-α/EHHADH pathway mainly regulated lipid metabolism, we investigated the fatty acid level in the MICA+ HCC cells. We found the increased lipid accumulation in the MICA+ HCC cells co-cultured with macrophages for 72 h (Figure S7b,c and Figure 4m). Collectively, MICA+ HCC cells increased fatty acid accumulation through the decreased PPAR-α/EHHADH signaling pathway.

### 3.5. The Increased FAO Level Induced Phenotypic Alteration in Macrophages

**Figure 5:**
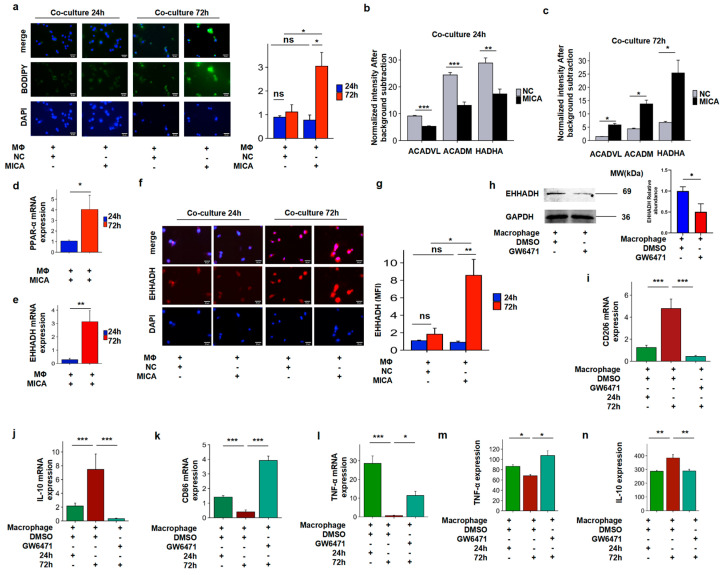
Co-culture of HCC cells with macrophage assays to verify the effect of FAO on phenotypic alteration in macrophages and the effect of FAO on macrophage alteration through the PPAR-α/EHHADH signaling pathway. (**a**) Representative BODIPY staining for co-cultured macrophages for 24 h and 72 h and associated statistical plots (*n* = 3). (**b**) The ACADVL, ACADM, and HADHA expression are shown in co-cultured macrophages after 24 h, respectively (*n* = 6). (**c**) The ACADVL, ACADM, and HADHA expression are shown in co-cultured macrophages after 72 h, respectively (*n* = 7). (**d**) The PPAR-α mRNA expression levels in macrophages co-cultured with MICA+ Huh-7 cells for 24 h and 72 h (*n* = 10). (**e**) The EHHADH mRNA expression levels in macrophages co-cultured with MICA+ Huh-7 cells for 24 h and 72 h (*n* = 8). (**f**) IF staining confirm increased EHHADH expression in macrophages co-cultured with MICA+ Huh-7 cells compared with NC cells for 72 h (*n* = 5). (**g**) Associated statistical plots confirm increased EHHADH expression in macrophages co-cultured with MICA+ Huh-7 cells compared with NC cells for 72 h (*n* = 5). (**h**) Western blotting confirm decreased EHHADH expression in macrophages treated with the PPAR-α inhibitor GW647 compared with DMSO. The statistically quantificational results are shown on the right (*n* = 3). The uncropped blots are shown in Supplementary File S1. (**i**) The CD206 mRNA expression levels in macrophages co-cultured with MICA+ Huh-7 cells for 24 h and 72 h after treated with GW6471 or DMSO are shown by qPCR (*n* = 8). (**j**) The IL-10 mRNA expression levels in macrophages co-cultured with MICA+ Huh-7 cells for 24 h and 72 h after treated with GW6471 or DMSO are shown using qPCR (*n* = 10). (**k**) The CD86 mRNA expression levels in macrophages co-cultured with MICA+ Huh-7 cells for 24 h and 72 h after treated with GW6471 or DMSO are shown using qPCR (*n* = 7). (**l**) The TNF-α mRNA expression levels in macrophages co-cultured with MICA+ Huh-7 cells for 24 h and 72 h after treated with GW6471 or DMSO are shown using qPCR (*n* = 9). (**m**,**n**) The TNF-α (*n* = 5) and IL-10 (*n* = 4) levels in supernatants from cultured macrophages were measured by ELISA. All images were taken at 400× magnification. *p*-value significance codes: * *p* < 0.05, ** *p* < 0.01, and *** *p* < 0.001.

Given the known metabolic crosstalk between tumor cells and macrophages mediated by free fatty acids (FFAs), which enhances FAO and induces M2-like polarization [48], we aimed to investigate whether the alteration in macrophages was induced by increased FAO levels from the FFAs in MICA+ HCC cells. Initially, we confirmed an increase in lipid accumulation in macrophages co-cultured with MICA+ HCC cells for 72 h compared to 24 h (Figure 5a and Figure S7d,e).

Since ACADVL, ACADM, and HADHA are key enzymes involved in the FAO pathway, assessing their expression would provide insights into FAO levels. Subsequently, we observed a decrease in FAO in macrophages co-cultured with MICA+ HCC cells for 24 h (Figure 5b). Conversely, FAO levels significantly increased in macrophages co-cultured with MICA+ HCC cells for 72 h (Figure 5c). Taken together, our findings suggest that MICA+ HCC cells induce alterations in macrophages through increased fatty acid accumulation and FAO levels.

We subsequently investigated the mechanism of FAO in macrophages. Our previous scRNA-seq data analysis revealed that EHHADH and PPAR-α were predominantly expressed in HCC cells (Figure 4b); however, an increase in EHHADH levels in TAMs with elevated FAO levels was observed [44]. Thus, we aimed to investigate whether the FAO of macrophages was also regulated by the PPAR-α/EHHADH pathway. As anticipated, the mRNA expression of EHHADH and PPAR-α was higher in macrophages co-cultured for 72 h compared to 24 h (Figure 5d,e). Furthermore, IF staining assays observed a significantly increased EHHADH protein expression in macrophages co-cultured for 72 h (Figure 5f,g). Subsequently, our WB analysis confirmed the decreased EHHADH protein expression in macrophages treated with the PPAR-α inhibitor GW6471 (Figure 5h). Importantly, blocking the PPAR-α/ EHHADH pathway with the PPAR-α inhibitor GW6471 resulted in a switch in the phenotype and function of macrophages (Figure 5i–n).

**Figure 6:**
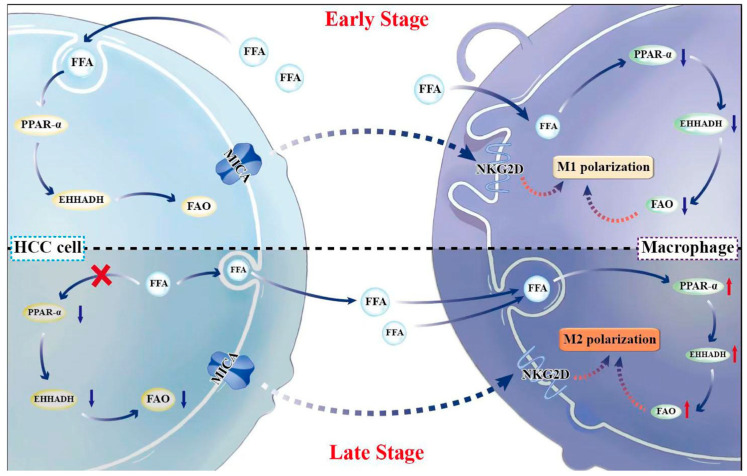
The summary of the findings in this study. In early-stage HCC, MICA expression in tumor cells and decreased PPAR-α/EHHADH level in macrophages induced M1-like macrophages infiltration. In late stage, the decreased EHHADH expression in MICA+ HCC cells suppressed FAO and increased FFA. The increased FFA was transported into macrophage to promote FAO level to induce M2-like polarization through PPAR-α/EHHADH pathway. ↑: increased expression; ↓: decreased expression.

In summary, the expression of MICA in tumor cells leads to the infiltration of M1-like macrophages in early-stage HCC in co-culture models. However, in the late stage in co-culture models, a decrease in EHHADH expression is observed in MICA+ HCC cells, resulting in increased FFAs. The increased FFAs are transported into macrophages, enhancing FAO levels and inducing M2-like macrophage polarization through the PPAR-α/EHHADH pathway (Figure 6).

## 4. Discussion

In this study, we have identified the functional regulation of FAO in HCC tumor cells and macrophages by the MICA-associated metabolic enzyme EHHADH and its upstream molecule PPAR-α. Our findings are consistent with previous studies that have demonstrated the ability of EHHADH to promote FAO [43,44,49]. FAO is a crucial process in lipid metabolism that is closely linked to the biological functions of both tumor cells and macrophages. Suppressed FAO has been shown to promote the aggressiveness of HCC cells [50]. FAO also provides energy for anti-inflammatory M2-like macrophages [44,51,52], indicating that FAO has diverse effects on HCC cells and macrophages.

Furthermore, we have discovered that EHHADH expression is decreased in HCC tumors and can serve as a biomarker for predicting the prognosis of patients with HCC, as supported by our biostatistical analyses. This finding is consistent with a previous study that identified EHHADH as a tumor suppressor gene in HCC tissues, with higher expression levels being associated with better prognosis [51]. We have also confirmed the decreased expression of EHHADH in our collected tumor tissues.

Tumor immune cells play an immunomodulatory role in TME, which is closely related to the immune escape of tumor cells and, thus, affects tumor progression [53]. Although EHHADH has been proved to be closely related to tumor prognosis [54], its relationship with tumor immune cells has not been studied in depth. Therefore, in order to better explore the relationship between EHHADH and tumor immune cells, we analyzed the TIMER database and found a significant association between EHHADH expression and the infiltration of five types of immune cells in tumors, namely B cells, CD8+ T cells, CD4+ T cells, neutrophils, and macrophages. Among these, TAMs have been shown to possess suppressive effects on antitumor immunity and promote tumor progression [55,56,57]. Additionally, our analyses using the cBioPortal platform have revealed a negative correlation between EHHADH expression and the levels of M1-like macrophage markers CD80 and CD86, while a positive correlation was observed with the M2-like macrophage marker CD206. These findings suggest a potential relationship between EHHADH expression and macrophage polarization. Furthermore, our qPCR and IHC experiments have provided results consistent with the biostatistical analyses, supporting the notion that increased EHHADH expression may lead to macrophage polarization towards an M2-like phenotype through the promotion of FAO.

In contrast, we have observed a positive correlation between MICA expression and the levels of CD80 and CD86, while a negative correlation was found with CD206. These findings suggest a potential association between MICA and macrophage phenotype, indicating that MICA and EHHADH may have opposing effects on macrophage polarization. Moreover, our co-culture experiments involving MICA+ HCC cells and macrophages have confirmed that MICA influences macrophage alteration through EHHADH.

Fatty acid metabolism plays a crucial role in various biological processes, including energy production, carbohydrate synthesis, and intracellular signaling regulation [58]. M2-like macrophages primarily rely on FAO for energy, whereas M1-like macrophages depend on glycolysis [59,60]. The different modes of energy supply have a significant impact on macrophage polarization and associated biological functions. Our study has demonstrated a negative correlation between MICA levels and EHHADH in HCC cells. Additionally, we have observed that the decreased EHHADH in HCC cells leads to increased FFA absorption by macrophages, thereby promoting FAO levels and enhancing M2-like macrophage polarization.

The previous study has demonstrated that increased FAO in TAMs leads to the conversion of a M2-like phenotype. Furthermore, inhibition of FAO has been shown to reverse the immunosuppressive activity of macrophages and inhibit HCC tumorigenesis [52]. Conversely, induction of glycolysis in macrophages promotes a reliance on glucose metabolism and subsequent conversion to a M1-like phenotype. The enhanced glucose utilization and increased glycolysis in macrophages have been associated with M1-like macrophage polarization, an increased M1/M2 ratio, remodeling of tumor immunity, and the exertion of antitumor effects in HCC [61]. Our findings are consistent with these previous studies. However, further investigations are required to explore the specific blockade of EHHADH and FAO in macrophages, as this will determine the phenotypic alterations and functional remodeling of macrophages, ultimately improving the effectiveness of antitumor immunity.

In brief, this study provides evidence that the MICA-related metabolic gene EHHADH has potential as a prognostic biomarker for HCC. It was observed that MICA induces macrophages to polarize towards a M1-like phenotype, resulting in pro-inflammatory effects during the early-stage HCC in co-culture models, which may have an antitumor effect. However, in late-stage HCC cells in co-culture models, MICA induces a decrease in EHHADH expression, leading to macrophage conversion to the anti-inflammatory M2-like phenotype, which may contribute to tumor progression. This conversion is facilitated by increased fatty acid utilization by macrophages, promoting FAO for energy production. However, due to the limitations of various factors, we were unable to further explore other possible mechanisms of action, which is also the limitation of this study. We look forward to more in-depth research to better understand this phenomenon.

In conclusion, EHHADH has become a potential therapeutic target for HCC. Combining the induction of MICA (pro-inflammatory effect) in cancer cells with the specific inhibition of EHHADH and FAO (anti-inflammatory effect) in macrophages may offer a beneficial therapeutic approach for HCC.

## 5. Conclusions

The metabolic gene EHHADH, which is associated with MICA, led to alterations in M2-like macrophages by promoting heightened fatty acid uptake and augmenting levels of FAO within macrophages.

## Figures and Tables

**Table 1:** Primer sequences.

Gene Name	Sequence of Primer
MICA	F: CACAGCGGGAATCACAGCACTC
	R: ATAGCAGCAGCAGCAACAGCAG
EHHADH	F: GTCAACGCGATCAGTACGAC
	R: CCTAGGAGCACTGAAGCCAC
PPAR-α	F: TCGGCGAGGATAGTTCTGGAAGC
	R: ACCACAGGATAAGTCACCGAGGAG
CD68	F: GTTCATCCAACAAGCAACAGCACTG
	R: CGGAGAGGGTGGAGGTGGTTC
CD86	F: GGAACCAACACAATGGAGAG
	R: AAACACGCTGGGCTTCATC
CD206	F: TCGGGTTTATGGAGCAGGTG
	R: TGAACGGGAATGCACAGGTT
GAPDH	F: GCACCGTCAAGGCTGAGAAC
	R: TGGTGAAGACGCCAGTGGA
TNF-α	F: TGCTCCTCACCCACACCAT
	R: GGAGGTTGACCTTGGTCTGGTA
IL-10	F: ATCCAAGACAACACTACTAA
	R: TAAATATCCTCAAAGTTCC
PPAR-γ	F: CCAGAAGCCTGCATTTCTGC
	R: GTGTCAACCATGGTCATTTCGTT

## Data Availability

The supporting data and materials in this research are available upon reasonable request to the corresponding author.

## Supplementary Materials

The following supporting information can be downloaded at: https://www.mdpi.com/article/10.3390/cancers17142365/s1, Figure S1: Enrichment analysis of target gene sets, EHHADH expression, and associated DEG function enrichment analysis in HCC; Figure S2: Prognostic value analysis of MICA in HCC and changes in proliferation and apoptosis of HCC cells overexpressing MICA; Figure S3: Correlation of EHHADH expression with different immune cell infiltration level; Figure S4: Correlation of EHHADH, MICA, and macrophage phenotype signature genes; Figure S5: The qPCR to verify correlation of MICA and EHHADH expression and macrophage infiltration in HCC; Figure S6: qPCR experimental validation of PPAR and EHHADH expression in HCC; Figure S7: Immunofluorescence assay and Oil Red O staining to verify the expression of EHHADH in MICA+HCC cells and macrophages; Table S1: Clinical information of five patients; Table S2: Cell markers; File S1: Full pictures of the Western blots.

## Abbreviations

AOD	Average optical density
BP	Biological process
cBioportal	cBio Cancer Genomics Portal
CC	Cellular component
CEG	Co-expression genes
CNV	Copy number variation
DAB	3,3′-diaminobenzidine
DAP10	DNAX-Activation Protein 10
DEG	Differentially expressed gene
EHHADH	Enoyl-CoA Hydratase And 3-Hydroxyacyl CoA Dehydrogenase
FAO	Fatty acid oxidation
FAS	Fatty acid synthesis
FBS	Fetal bovine serum
FFA	Free fatty acid
GEPIA	Gene Expression Profiling Interaction Analysis
GO	Gene Ontology
HCC	Hepatocellular carcinoma
HPA	The Human Protein Atlas
IHC	Immunohistochemistry
IL	Interleukin
KEGG	Kyoto Encyclopedia of Genes and Genomes
LPS	Lipopolysaccharide
MF	Molecular function
MHC	Major histocompatibility complex
MICA	Major histocompatibility complex class I polypeptide-related sequence A
MRGs	Metabolism-related genes
NC	Negative control
NKG2D	Natural killer group 2, member D
PCA	Principal component analysis
PCs	Principal components
PMA	Phorbol 12-myristate 13-acetate
PPP	Pentose phosphate pathway
qRT-PCR	Quantitative real-time PCR
ROC	Receiver operating characteristic
scRNA-seq	Single-cell RNA sequence
TAM	Tumor-associated macrophages
TCA	Tricarboxylic acid cycle
TCGA	The Cancer Genome Atlas
TIMER	Tumor IMmune Estimation Resource
TME	Tumor microenvironment
