# Supplementary material for: MICA+ Tumor Cells Modulate Macrophage Phenotype and Function via PPAR/EHHADH-Mediated Fatty Acid Metabolism in Hepatocellular Carcinoma (HCC)
Source: Cancers (Basel). 2025 Jul 16;17(14):2365. doi: 10.3390/cancers17142365 (PMC12293600; doi:10.3390/cancers17142365)
Supplement: Supplementary file 1 [file cancers-17-02365-s001.zip › cancers-3694736-supplementary/Figures.pdf]

MICA+ Tumor Cells Modulate Macrophage Phenotype and Function via PPAR/EHHADH-Mediated Fatty Acid Metabolism in Hepatocellular Carcinoma（HCC）

Jingquan Huang [1,†], Yumeng Teng [1,†], Peng Yan [1], Yan Yang [1], Shixun Lin [1], Qiulin Wu [1], Qiang Du [2], Xicai Li [1],
Ming Yao [1], Jianjun Li [1], Yubin Huang [1], Xiaoyong Cai [1], David A. Geller [2] and Yihe Yan [1]

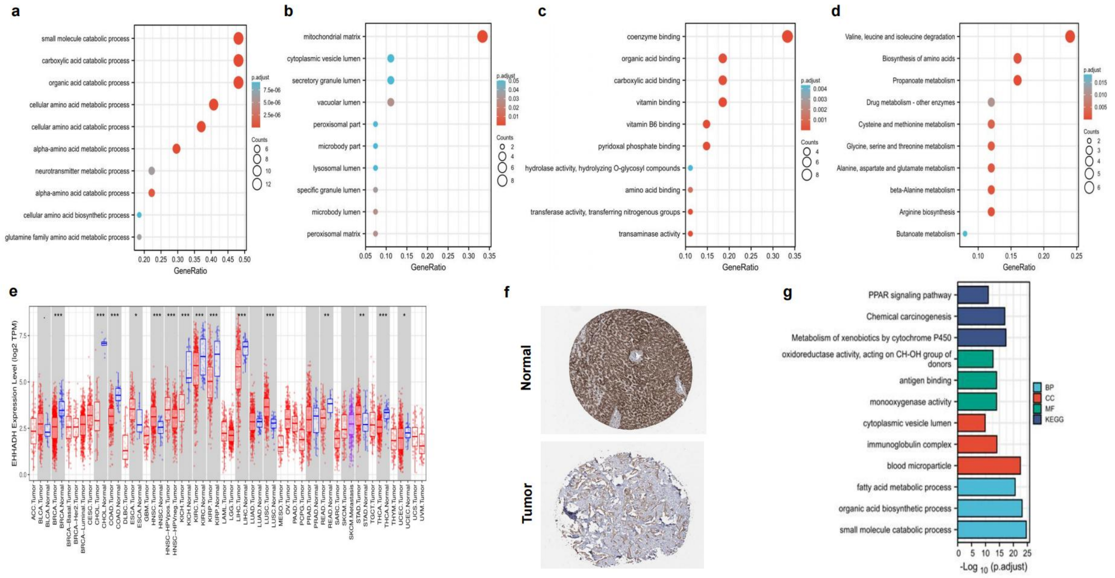

Figure S1. Enrichment analysis of target gene sets, EHHADH expression and associated DEG function enrichment analysis in HCC. **A-C** GO functional enrichment analysis of gene sets shows BP, CC and MF. **D** KEGG shows pathway enrichment analysis. The X-axis represents proportion of genes, and Y-axis represents different enrichments. Different colors represent different properties (p-values), and different sizes represent the number of genes. **E** EHHADH mRNA expressions are showed in pan-cancer. **F** IHC staining for EHHADH from HPA. **G** Functional enrichment of DEGs from high and low EHHADH expression in HCC is showed. *P*-value Significant Codes: * $P < 0.05$, ** $P < 0.01$, *** $P < 0.001$.

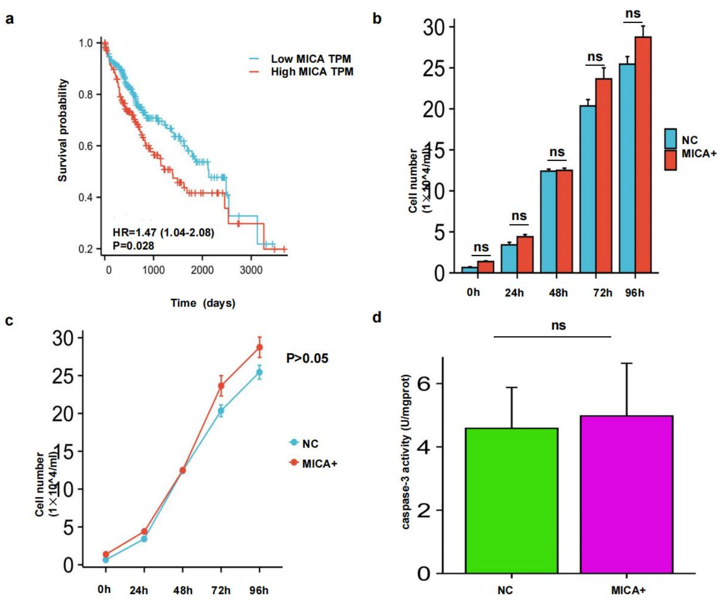

Figure S2. Prognostic value analysis of MICA in HCC and changes in proliferation and apoptosis of HCC cells overexpressing MICA. **A** K-M survival analysis of MICA expression in HCC. **B** Histogram of cell proliferation in MICA+ Huh-7 cells vs. control cells. **C** Line graph of cell proliferation in MICA+ Huh-7 cells vs. control cells (n=4). **D** Apoptosis analysis in MICA+ Huh-7 cells vs. control cells (n=3).

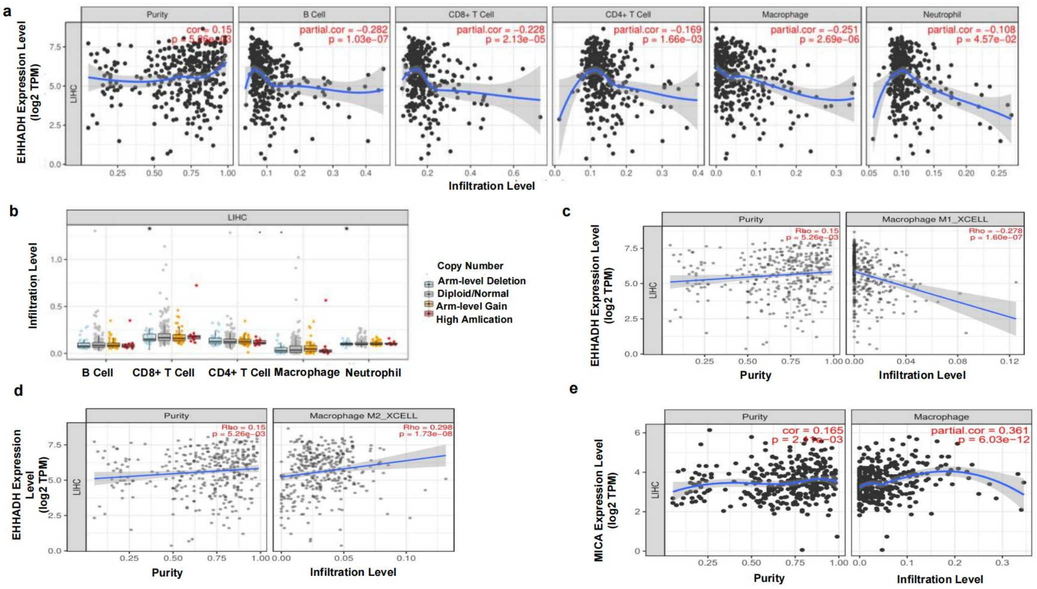

Figure S3. Correlation of EHHADH expression with different immune cell infiltration level. **A** Correlation of EHHADH with tumor purity and infiltration level of B cells, CD8+ T cells, CD4+ T cells, macrophages and neutrophils. **B** Relationship between CNV of EHHADH gene and different immune cells infiltration level. **C** Correlation of EHHADH mRNA expression with M1 macrophage infiltration. **D** Correlation of EHHADH with M2 macrophage infiltration. **E** Correlation of MICA with macrophage infiltration. *P*-value Significant Codes: * P < 0.05, ** P < 0.01, *** P < 0.001.

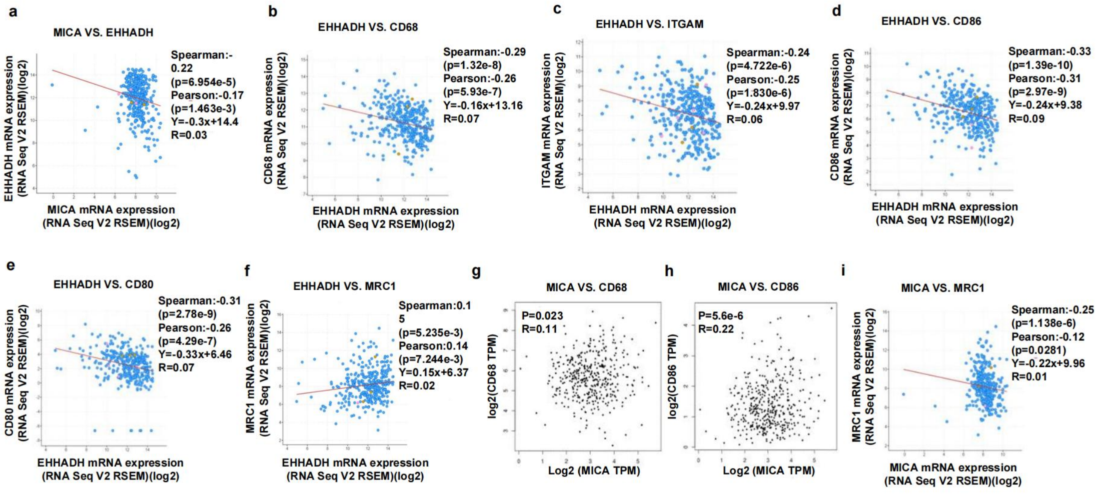

Figure S4. Correlation of EHHADH, MICA and macrophage phenotype signature genes. **A-F** Correlation of EHHADH mRNA expression with MICA, CD68, ITGAM, CD86, CD80, and MRC1 level. **G-I** Correlation of MICA mRNA expression with CD68, CD86, and MRC1 level.

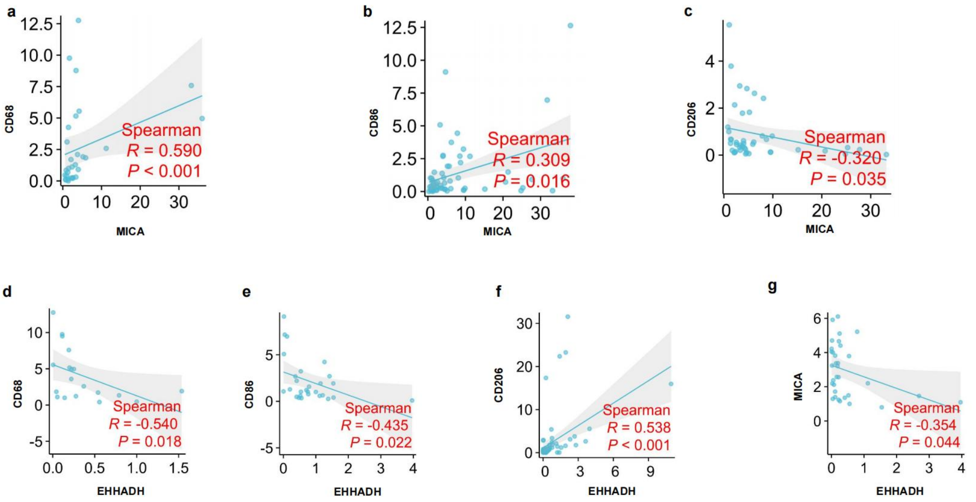

Figure S5. The qPCR to verify correlation of MICA & EHHADH expression and macrophage infiltration in HCC. **A** Correlation of MICA mRNA expression with CD68 mRNA level is showed in tumor and background liver tissues from HCC patients (n=15). **B** Correlation of MICA mRNA expression with CD86 mRNA level is showed in tumor and background liver tissues from HCC patients.(n=30). **C** Correlation of MICA mRNA expression with CD206 mRNA level is showed in tumor and background liver tissues from HCC patients (n=23). **D** Correlation of EHHADH mRNA expression with CD68 mRNA level is showed in tumor and background liver tissues (n=10). **E** Correlation of EHHADH mRNA expression with CD86 mRNA level is showed in tumor and background liver tissues (n=15). **F** Correlation of EHHADH mRNA expression with CD206 mRNA level is showed in tumor and background liver tissues (n=24). **G** Correlation of EHHADH mRNA expression with MICA mRNA level is showed in tumor and background liver tissues (n=17).

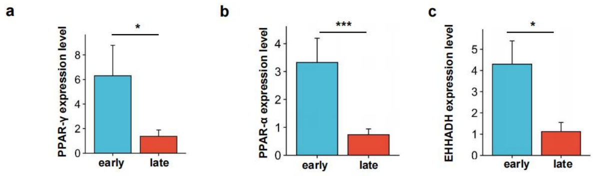

Figure S6. qPCR experimental validation of PPAR and EHHADH expression in HCC. **A-C** qPCR assay verified the expression of PPAR-γ, PPAR-α, and EHHADH mRNA in early stage HCC compared to late stage (n=21).

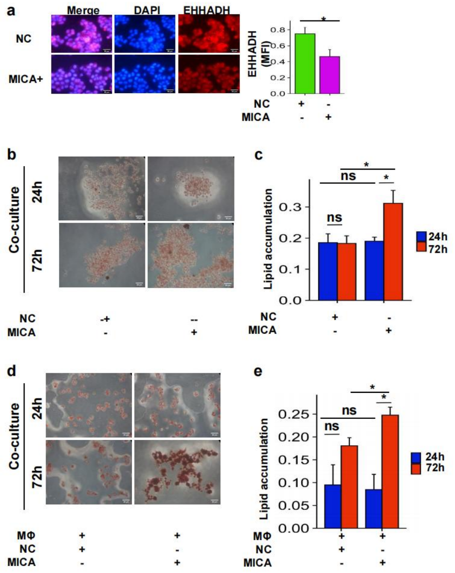

Figure S7. Immunofluorescence assay and oil red O staining to verify the expression of EHHADH in MICA+HCC cells and macrophages. **A** Representative immunofluorescence staining for EHHADH in MICA+ Huh-7 cells compared to NC+ Huh-7 cells and statistically quantificational results (n=5). **B** Representative oil red O staining for co-cultured Huh-7 cells for 24h and 72h. **C** Associated statistical plots of oil red O staining for co-cultured Huh-7 cells for 24h and 72h and associated statistical plots (n=5). **D** Representative oil red O staining for co-cultured macrophages for 24h and 72h. **E** Associated statistical plots of oil red O staining for co-cultured macrophages for 24h and 72h (n=5).
