# Supplementary material for: MICA+ Tumor Cells Modulate Macrophage Phenotype and Function via PPAR/EHHADH-Mediated Fatty Acid Metabolism in Hepatocellular Carcinoma (HCC)
Source: Cancers (Basel). 2025 Jul 16;17(14):2365. doi: 10.3390/cancers17142365 (PMC12293600; doi:10.3390/cancers17142365)
Supplement: Supplementary file 1 [file cancers-17-02365-s001.zip › cancers-3694736-supplementary/Tables.pdf]

Table S1. Clinical information of five patients

Patients	Viral infection	Stages
HCC01	HBV	I
HCC02	HBV	I
HCC06	HBV	IIIA
HCC09	HBV	IV
HCC10	HBV	IV

Table S2. Cell Marker

Cell	Marker
Endothelial cells	PECAM1,CDH5
Hepatic stellate cells	ACTA2,PDGFRB
Malignant cells	AFP,GPC3
Normal epithelial cells	EPCAM,KRT19
NK cells	KLRB1,KLRD1
T cells	CD3D,CD4,CD8A
M1-macrophages	CD68,CD86
M2-macrophages	CD68,CD206
Dendritic cells	CD1C
B cells	CD79A
