# Supplementary figures and images for: MICA+ Tumor Cells Modulate Macrophage Phenotype and Function via PPAR/EHHADH-Mediated Fatty Acid Metabolism in Hepatocellular Carcinoma (HCC)
Source: Cancers (Basel). 2025 Jul 16;17(14):2365. doi: 10.3390/cancers17142365 (PMC12293600; doi:10.3390/cancers17142365)

Figure 3A

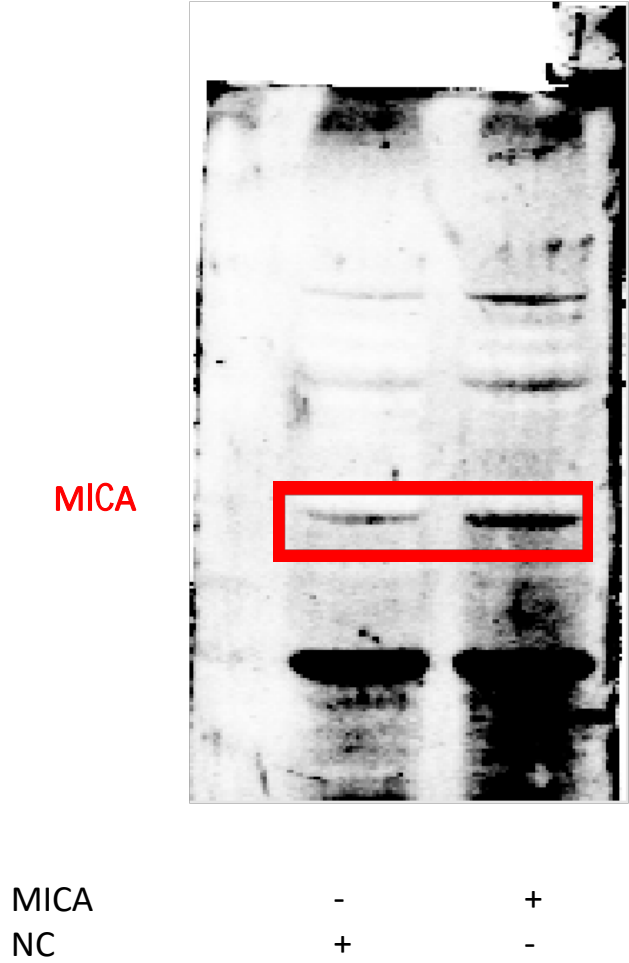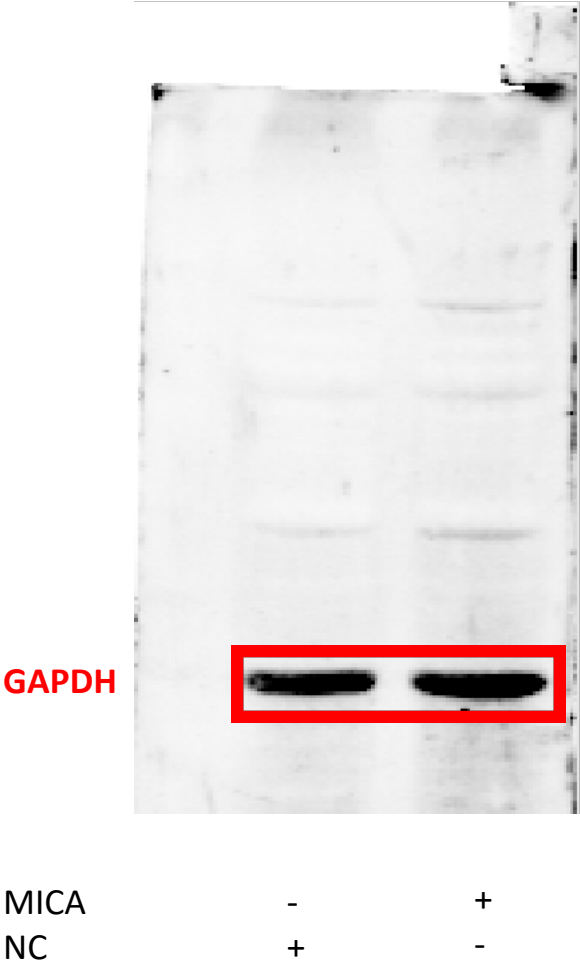

Figure 4I

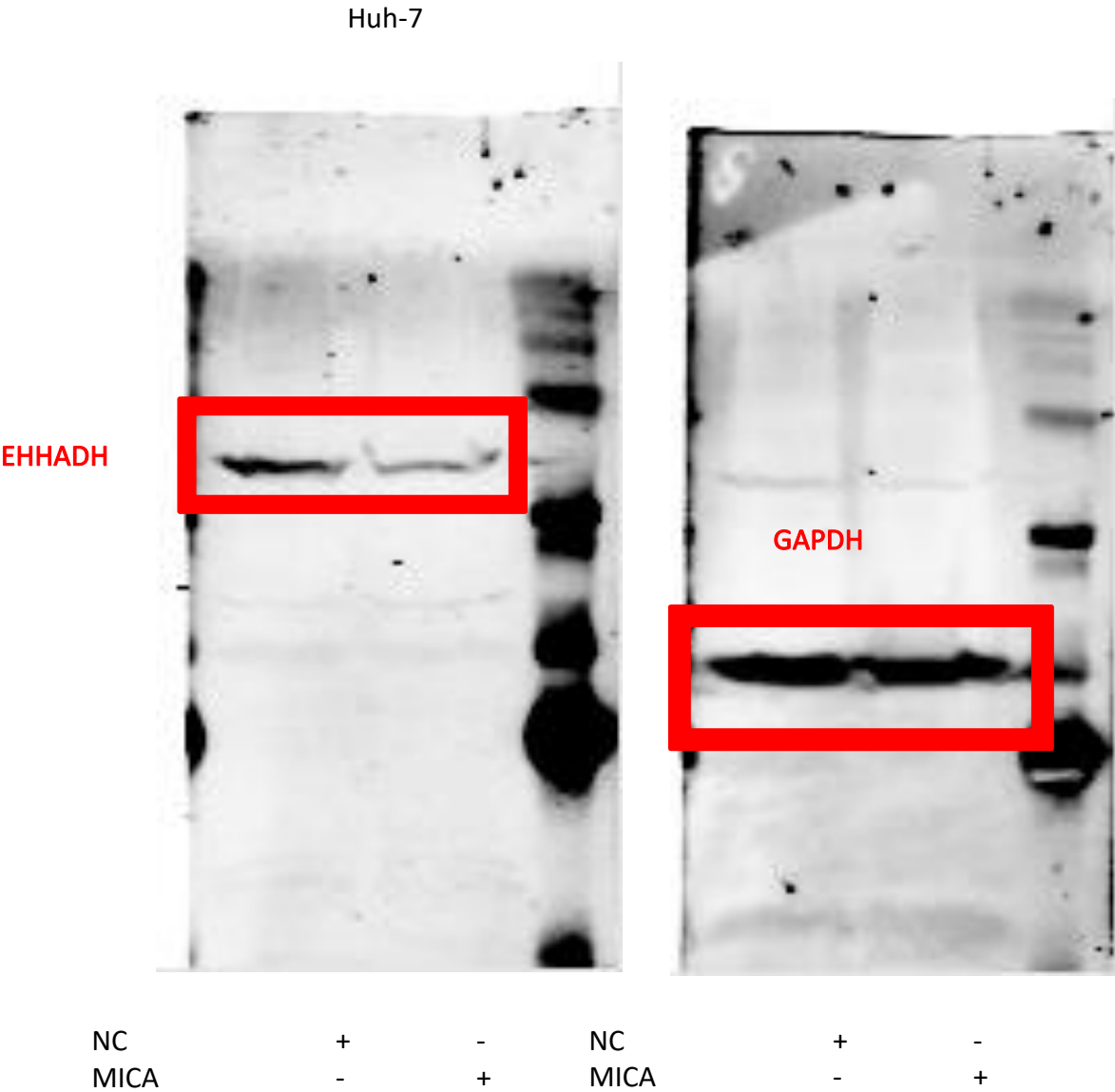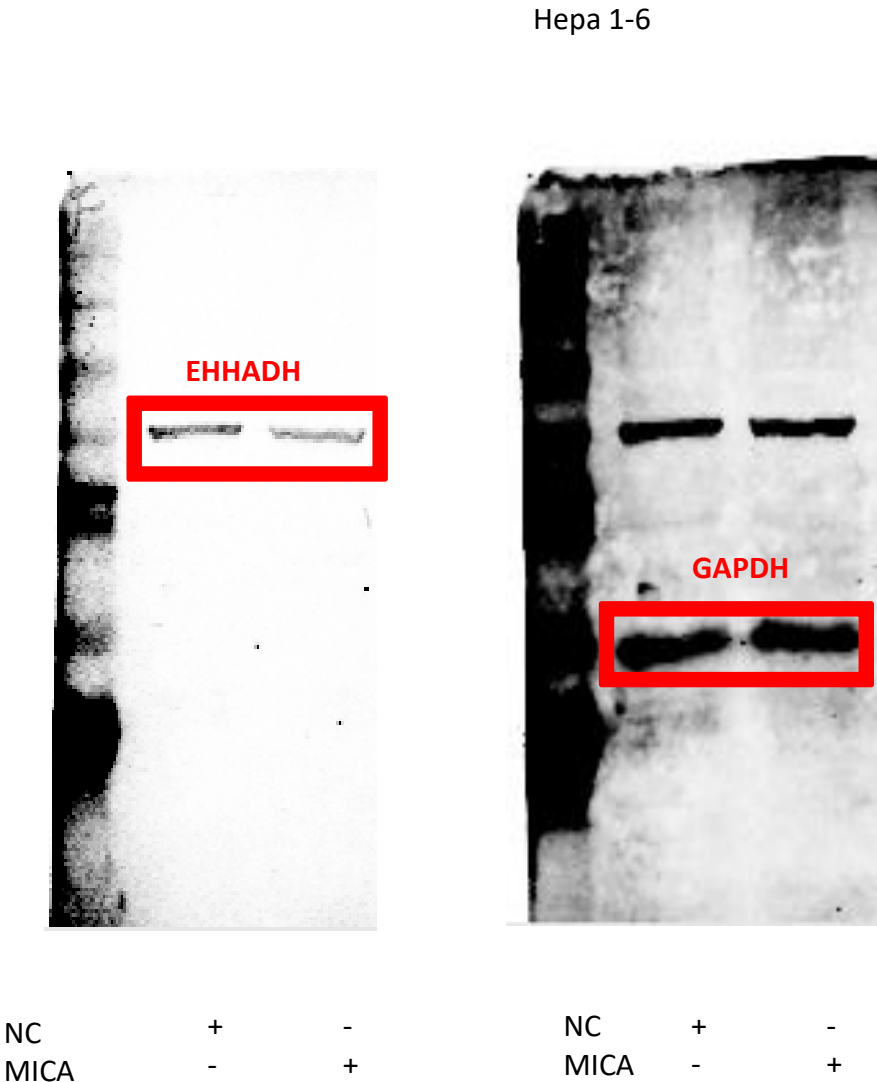

Figure 4J

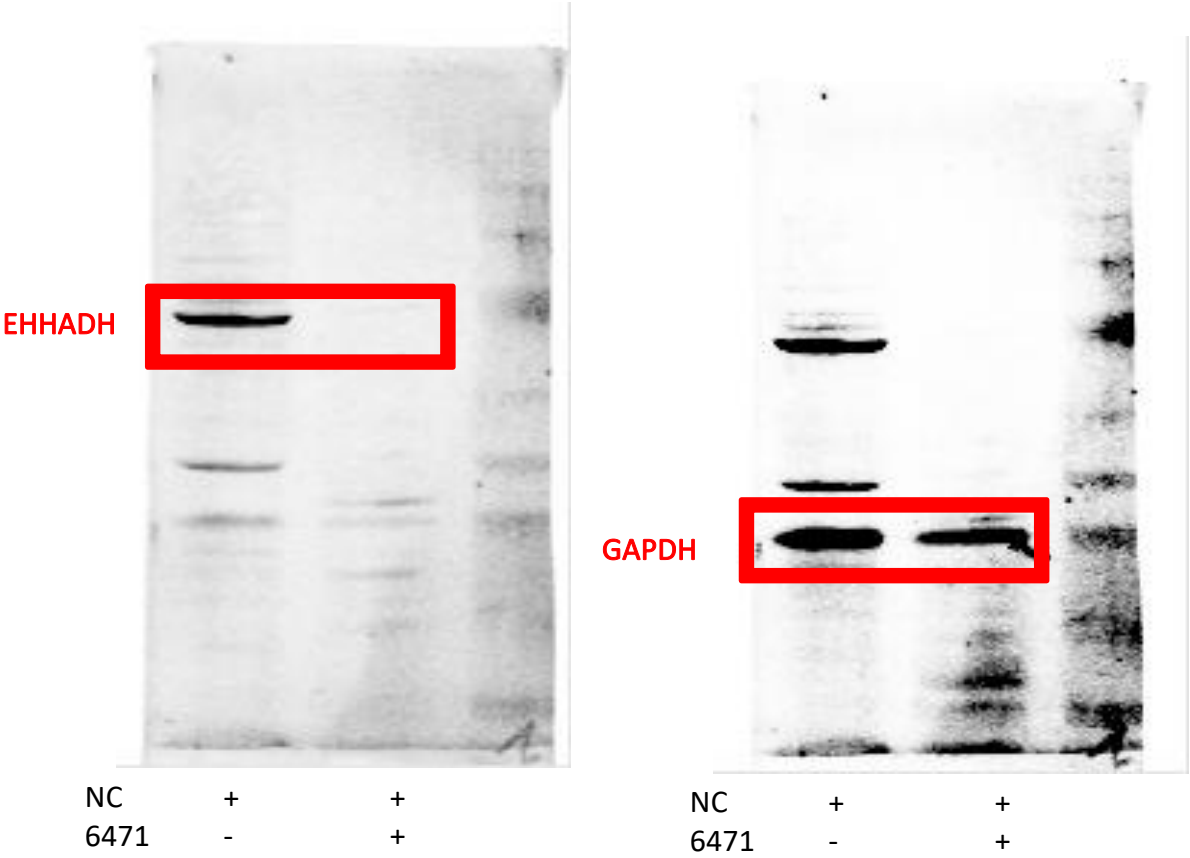

Figure 4K

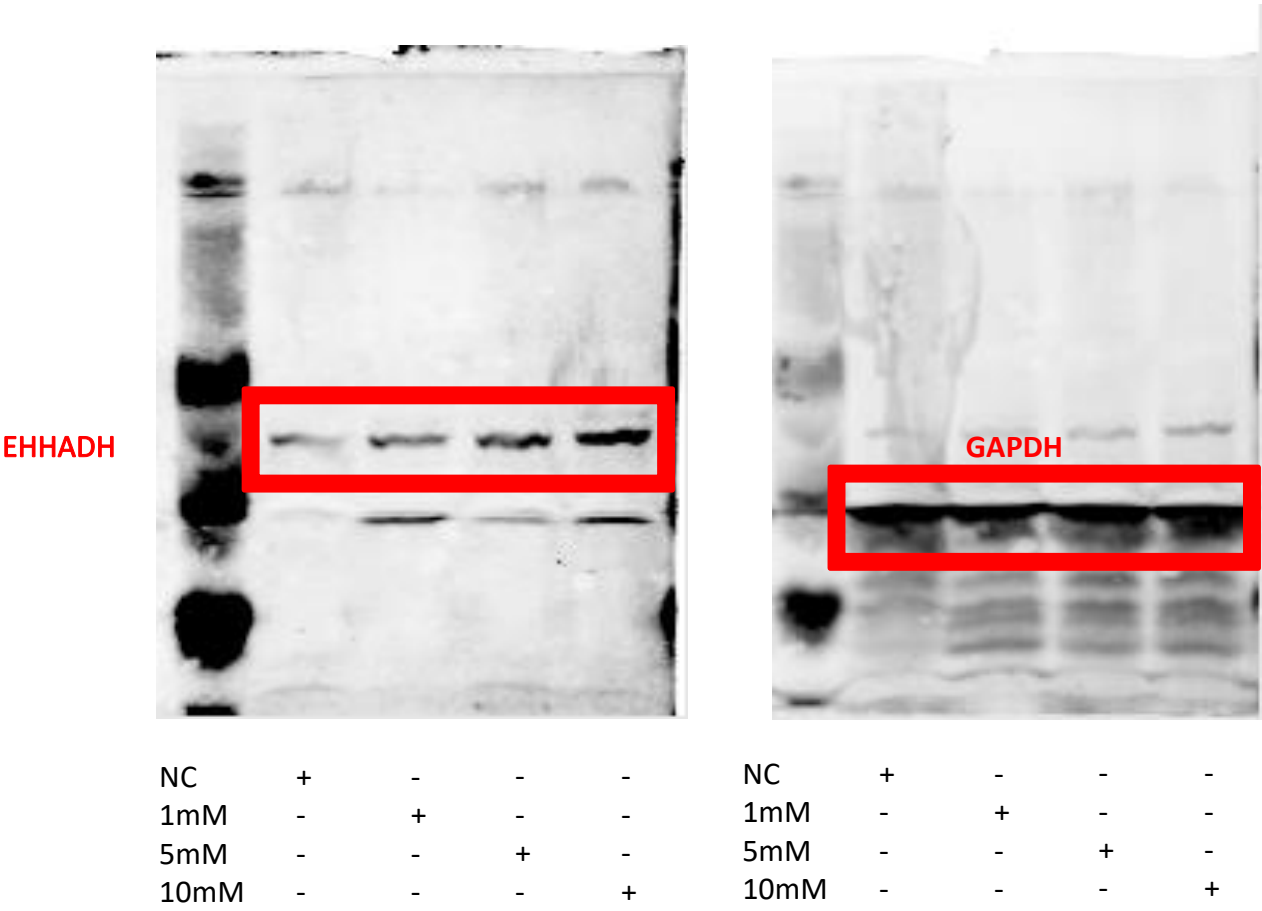

Figure 4L

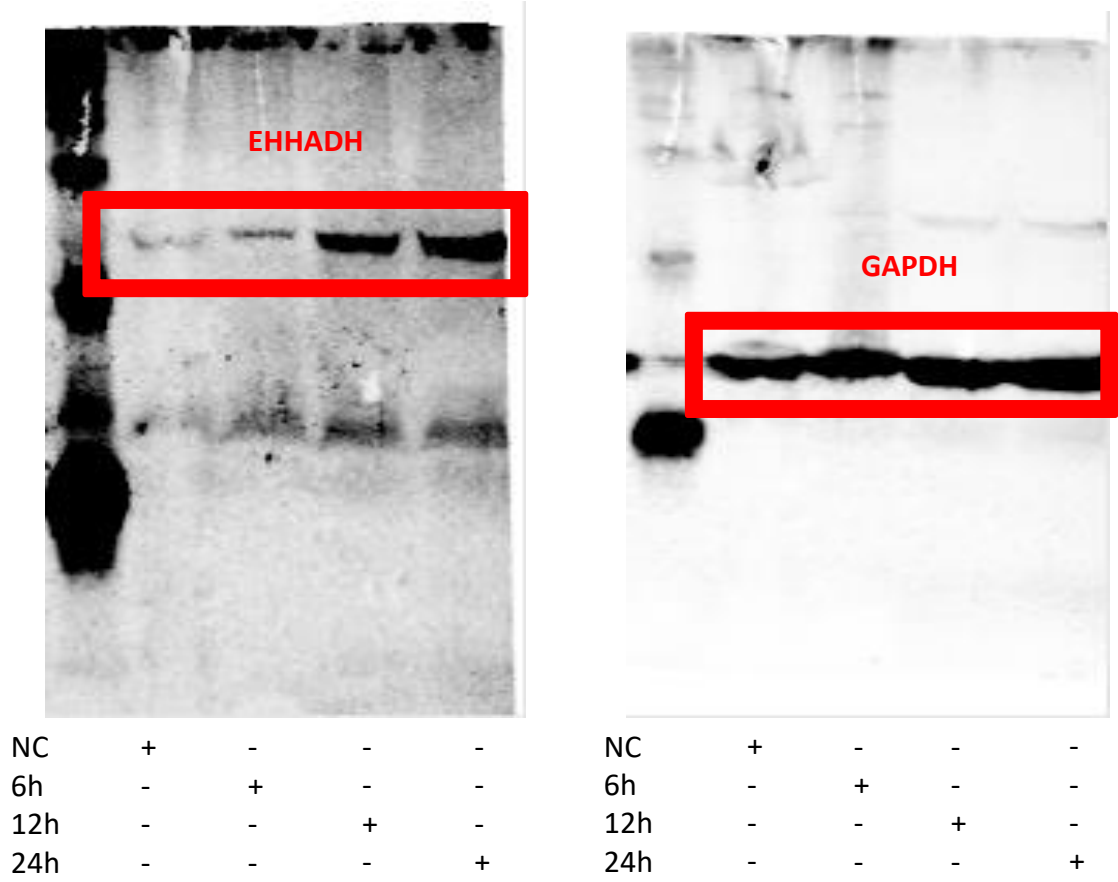

Figure 5H

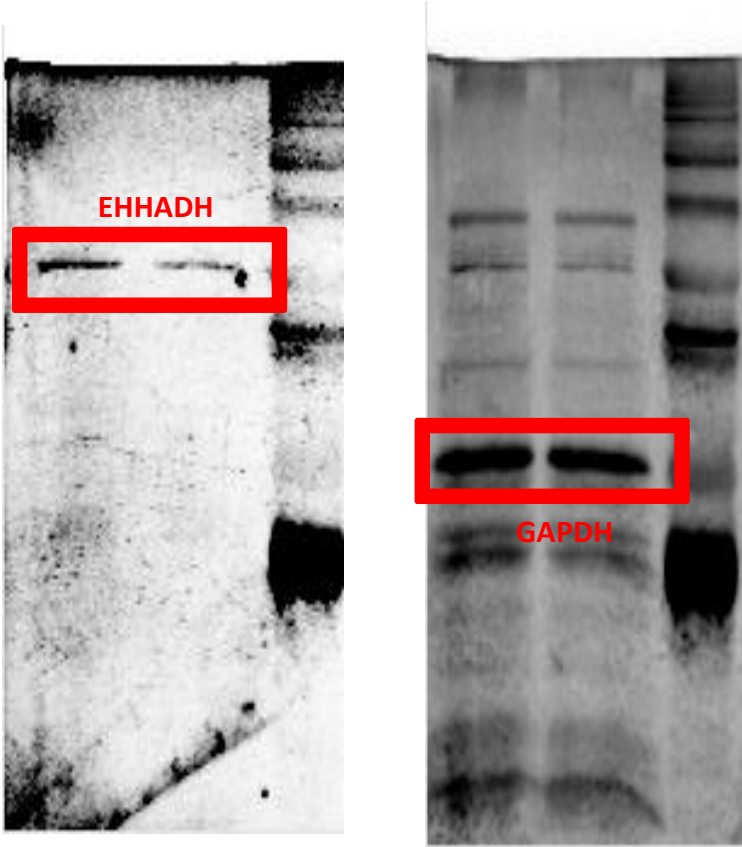

|            |   |   |            |   |   |
|------------|---|---|------------|---|---|
| Macrophage | + | + | Macrophage | + | + |
| DMSO       | + | - | DMSO       | + | - |
| GW6471     | - | + | GW6471     | - | + |

Supplement: Supplementary file 1 [file cancers-17-02365-s001.zip › cancers-3694736-supplementary/File S1-original-images.pdf]
